# Rigorous Science: a How-To Guide

**DOI:** 10.1128/mBio.01902-16

**Published:** 2016-11-08

**Authors:** Arturo Casadevall, Ferric C. Fang

**Affiliations:** a Department of Molecular Microbiology and Immunology, Johns Hopkins Bloomberg School of Public Health, Baltimore, Maryland, USA; b Departments of Laboratory Medicine and Microbiology, University of Washington School of Medicine, Seattle, Washington, USA

## Abstract

Proposals to improve the reproducibility of biomedical research have emphasized scientific rigor. Although the word “rigor” is widely used, there has been little specific discussion as to what it means and how it can be achieved. We suggest that scientific rigor combines elements of mathematics, logic, philosophy, and ethics. We propose a framework for rigor that includes redundant experimental design, sound statistical analysis, recognition of error, avoidance of logical fallacies, and intellectual honesty. These elements lead to five actionable recommendations for research education.

## EDITORIAL

Rigor is a prized quality in scientific work. Although the term is widely used in both scientific and lay parlance, it has not been precisely defined (1). Rigor has gained new prominence amid concerns about a lack of reproducibility in important studies (2, 3), an epidemic of retractions due to misconduct (4), and the discovery that the published literature is riddled with problematic images (5). Insufficient rigor may be slowing the translation of basic discoveries into tangible benefits (6, 7). New initiatives aim to understand deficiencies in scientific rigor and to make research more rigorous (8–10). Here, we consider the meaning of rigorous science and how it can be achieved.

The word rigor is derived from an old French word, “*rigueur*,” meaning strength and hardness (11). In scientific vernacular, the underlying concept of strength resonates in the expressions “hard data” and “solid work” used to convey a sense of reliable and trustworthy information. In common usage, the word “rigor” has evolved to mean the quality of being exact, careful, or strict (12). Although the words “exact” and “careful” also apply to science, additional definition is needed since practicing rigorous science means more than mere exactness and care in experimental design. An experiment in which all components were exact in their proportions and the procedures carefully executed would still not be considered rigorous in the absence of appropriate controls. Hence, the definition of scientific rigor requires a deeper exploration than can be provided by simple perusal of the dictionary.

The scientific literature adds surprisingly little to our understanding of rigor, with the term almost always used without definition, as if its meaning is self-evident. The NIH has recently defined scientific rigor as “the strict application of the scientific method to ensure robust and unbiased experimental design, methodology, analysis, interpretation and reporting of results” including “full transparency in reporting experimental details so that others may reproduce and extend the findings” (13). While we credit the NIH for providing a starting point for discussion, we find the NIH definition of rigor to be both excessively wordy and disconcertingly vague, as well as complicated by an insistence on transparency and reproducibility, which may be desirable but are arguably separate from rigor.

## A WORKING DEFINITION OF SCIENTIFIC RIGOR

We suggest that rigorous science may be defined as theoretical or experimental approaches undertaken in a way that enhances confidence in the veracity of their findings, with veracity defined as truth or accuracy. Rigorous science could be entirely theoretical, as exemplified by a thought experiment used to illustrate a principle, such as Schrödinger’s cat or Maxwell’s demon in physics, or entirely experimental, as illustrated by Cavendish’s measurement of the gravitational constant at the end of the 18th century. However, in the biomedical sciences, most research has both theoretical and experimental aspects.

## A PENTATEUCH FOR SCIENTIFIC RIGOR

Different fields vary in the level of uncertainty that they are willing to accept with regard to conclusions. Certainty in science is often couched in terms of the probability that the null hypothesis may be rejected, which in turn depends on the methodologies employed. For example, the Higgs boson was announced when physicists were certain to “five sigma” or a *P* value of 3 × 10^−7^ (14). In contrast, many biological and medical studies accept a *P* value of 0.05, although more stringent criteria have been advocated (15). Does this make physics more rigorous than biology? Not necessarily—differences in the complexity of physical and biological phenomena as well as limitations in methodology determine the level of certainty that is practically achievable in these disciplines. Hence, a definition of rigorous science cannot rely on strict and arbitrary levels of certainty.

**FIG 1:**
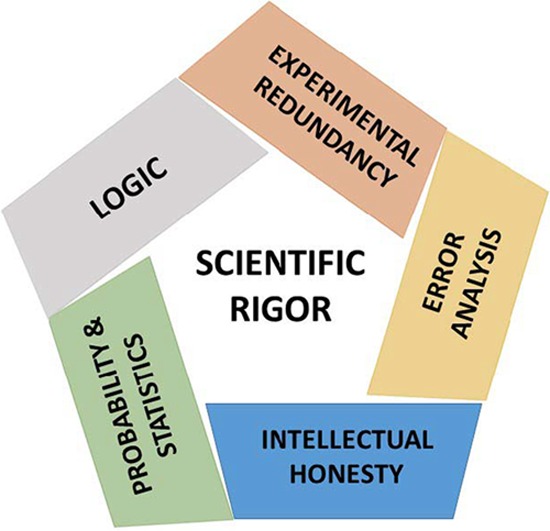
A Pentateuch for improving rigor in the biomedical sciences.

Traditional Chinese philosophy, Hinduism, Islam, and Judaism are each founded on five elements, pillars, or sacred texts. In Judaism, the first five books of the Hebrew bible are collectively referred to as the Pentateuch. Here, we humbly propose a Pentateuch for scientific rigor (Fig. 1).

### (i) Redundancy in experimental design.

Good laboratory practices include proper controls, dose-response studies, determination of time courses, performance of sufficient replicates, and corroboration of major findings using independent experimental approaches and methods. It is important to establish whether a finding is generalizable, using a variety of cell types or species. New findings should lead to new predictions, which can in turn be experimentally tested. Experimental confirmation of predictions provides added assurance that the original findings are valid. Like rigor, redundancy is a multidimensional quality composed of many elements (Table 1). Redundancy in experimental design can enhance confidence in experimental results.

**TABLE 1:** Some elements of scientific redundancy

Element^a^	Description and implementation
Replication	Carry out independent replicates, which provide information regarding the replicability and variability of an observation. This in turn influences the interpretation of the magnitude of the observed effects and the sample size required for statistical significance.
Validation	Validate the observation by an independent methodology. For example, the assignment of a protein on an immunoblot can be validated by immunoprecipitation using different antibodies, and the differential abundance of an mRNA by microarray or transcriptome sequencing can be validated by reverse transcription-quantitative PCR. This element also applies to purifications, which should use at least two independent methodologies. For example, chromatography can be complemented with differential sedimentation, electrofocusing, precipitation, etc. The element of validation is particularly important for experimental components such as antibodies and cell lines.
Generalization	Explore the generalizability of the findings. For example, in microbiological studies the use of different strains, cell lines, reagents, media, experimental conditions, etc., can be used to ascertain the generalizability of the finding. Findings that are generalizable are more likely to be robust.
Perturbation	Define the conditions under which the observation occurs by perturbing the system. For example, before reporting a biochemical observation, perturb the system by changing the pH or the ionic strength of the experimental conditions. Knowledge of the perturbation boundaries introduces redundancy since it inevitably includes replication and generalization and reveals the degree of resiliency, which in turn enhances the likelihood of replication.
Consistency	Determine whether the various observations that define a scientific study are internally consistent. Although internal consistency does not necessarily imply validity, its absence may suggest the presence of uncontrolled variables.

^a^ This list of elements is not exhaustive. The examples are provided to illustrate the principle of redundancy in experimental design. We note that some of these elements are interrelated and thus not independent. For example, any effort to validate or generalize a finding also involves replication. However, the elements listed are sufficiently distinct as to be considered independently when analyzing the redundancy of a scientific study.

### (ii) Sound statistical analysis.

The progression of biology from a qualitative science focused on classification to a quantitative science based on numerical data represents a triumph of the discipline. However, this means that biologists must become conversant in the principles of probability and statistics. Attention to power calculations and other statistical considerations is essential for a more rigorous approach to science. A determination of certainty is not enough—the size of an observed effect is crucial. A large effect is more likely to be important and perhaps more reproducible as well.

### (iii) Recognition of error.

Error is pervasive in research (16), and any experimental technique ranging from simple pipetting to crystallographic analysis is subject to error. Errors can be random or systematic and tend to propagate with multiple experimental steps. Random errors may occur during any experimental procedure, are unpredictable, and can be estimated by performing replicates. Systematic errors tend to be associated with scientific instruments but can also be introduced by the use of impure reagents, contaminated cell lines, etc. A rigorous approach to science must include a full appreciation of potential sources of error and of how error can affect experimental results and the incorporation of processes to authenticate key reagents and resources. In engineering, a sensitivity analysis examines how uncertainty or error in various parameters can influence the output of a system. Applying the principles of sensitivity analysis to scientific experiments can reveal the likelihood that a given result is true as well as identify potential sources of error. Results that remain robust despite variance in experimental conditions are more likely to be valid.

### (iv) Avoidance of logical traps*.*

Logical traps and fallacies lurk everywhere in experimental science, especially in the interpretation of results. The list of logical fallacies that can befall an investigator is lengthy and includes confirmation bias, congruence bias, affirming the antecedent, denying the antecedent, base-rate fallacy, etc. A bedrock principle of science is the possibility of falsification—confirmatory evidence cannot prove an assertion, but contradictory evidence may disprove it (17). Given that confirmatory evidence is not conclusive, scientists can enhance the rigor of their work by systematically challenging and attempting to falsify their hypotheses. Avoiding logical fallacies is therefore essential for a rigorous approach to science, but doing so requires training in critical thinking and avoiding illogical thought patterns that often come naturally to humans.

### (v) Intellectual honesty.

Intellectual honesty is a mindset that encompasses diverse concepts ranging from ethics to proper scientific practice (18). Intellectual honesty is synonymous with objectivity, an essential requirement for scientific rigor. Acknowledgment of nagging details that do not fit with one’s hypothesis is often the first step to a new understanding and a better hypothesis. Implicit in the acknowledgment of earlier work is the need to reconcile one’s observations with those made by others. Corroboration by independent researchers can enhance the confidence of the scientific community that a scientific finding is valid.

As illustrated by these five principles, scientific rigor is multifaceted. No single criterion can define it. Even the most careful experimental approach is not rigorous if the interpretation relies on a logical fallacy or is intellectually dishonest. On the other hand, the principles of rigor can be synergistic, as when a logical approach and the awareness of error lead to greater purposeful redundancy in experimental design.

## RIGOR AND REPRODUCIBILITY

Rigorous scientific practices enhance the likelihood that the results generated will be reproducible. However, reproducibility is not an absolute criterion of rigorous science. One might rigorously characterize the mass, composition, and trajectory of a comet that collides with the sun, but those measurements could never be reproduced since each comet is unique. Nevertheless, improvements in scientific rigor are likely to improve reproducibility.

## ENHANCING RIGOR IN BIOMEDICAL RESEARCH TRAINING

The five principles outlined above provide a road map for increasing rigor in the biomedical sciences (Fig. 1). An obvious step is to strengthen didactic training in experimental design, statistics, error analysis, logic, and ethics during scientific training. We have previously called for improvements in graduate education, including areas of philosophy such as logic, as well as in probability and statistics (19). A recent survey of over 1,500 scientists found that there is considerable support for such reforms (20). However, with the exception of statistics, none of these disciplines are formally taught in the training of scientists, and even statistics is not always a curricular requirement (21). Reforming scientific education will be a major undertaking since most academic faculty lack the necessary background to teach this material. A multifaceted approach would include greater attention in the laboratory environment and improvements in peer review and constructive criticism, as well as formal didactics. This would need to be accompanied by a cultural change in the scientific enterprise to reward rigor and encourage scientists to recognize, practice, and promote it. To initiate the process, we make the following five actionable recommendations.

### (i) Develop didactic programs to teach the elements of good experimental practices.

Most biomedical scientists currently learn the basics of scientific methods and practice in their graduate and postdoctoral training through a guild-like apprenticeship system in which they are mentored by senior scientists. Although there is no substitute for good mentorship, there is no guarantee that mentors themselves are well trained. Furthermore, there is no guarantee that such individualized training will provide the basic elements of good experimental science. Today, training programs seldom include didactic programs to teach good research practices. Such courses could ensure a baseline background for all trainees.

### (ii) Require formal training in statistics and probability for all biomedical scientists.

As the biological sciences have become increasingly quantitative, they have become increasingly dependent on mathematical tools for all aspects of experimental design, implementation, and interpretation. Although the use of statistical tools has become widespread in biomedical research, these tools are often misused. Some authorities have gone so far as to issue a warning on the use of *P* values (22, 23). Consequently, there is a need for more formal mathematical training in the biomedical sciences despite the fact that some scientists many not be naturally inclined to such fields, a recommendation made previously by us and others (19, 21).

### (iii) Refocus journal club discussion from findings to methods and approaches*.*

Journal clubs are invaluable training formats that allow discussions of science in the context of a specific publication. However, articles selected for journal club discussions are often the flashiest papers from high-impact journals, which limit article length and truncate descriptions of methodology. Journal club sessions that focus on limitations of methodology, appropriateness of controls, logic of conclusions, etc., could provide a recurring venue for discussions of what constitutes scientific rigor.

### (iv) Develop continuing education materials for biomedical sciences.

Fields in which basic information is changing rapidly, such as medicine, require continuing education to maintain competence. Science is arguably one of the most rapidly changing areas of human endeavor, and the biological sciences have experienced a revolution since the mid-20th century. Fields could develop continuing education materials for scientists that would allow them to gain proficiency in the latest techniques. Guidelines for the practice of science, analogous to those used for clinical practice and publication ethics, could help to establish standards that promote rigor.

### (v) Develop teaching aids to enhance the quality of peer review.

The scientific process is critically dependent on peer review in publication and grant funding. Remarkably, scientists are recruited into peer review activities without any training in this process, and the results can be uneven. As peer review is a process, it is amenable to study in order to identify best practices that can be promulgated to educate and improve reviewer performance. Strengthening peer review will help to ensure the quality of scientific information.

Enhancing scientific rigor can increase the veracity and reproducibility of research findings. This will require a tightening of standards throughout the scientific progress from training to bench science to peer review. Today’s reward system in biomedical research is primarily based on impact (24), and impactful work is highly rewarded regardless of its rigor. Consequently, a scientist may obtain greater rewards from publishing nonrigorous work in a high-impact journal than from publishing rigorous work in a specialty journal. Perhaps, it is time to rethink the value system of science. Prioritizing scientific rigor over impact would help to maintain the momentum of the biological revolution and ensure a steady supply of innovative, reliable, and reproducible discoveries to translate into societal benefits. Perhaps, it will even become *de rigueur*.
